# Social anxiety mediates workplace incivility and work engagement

**DOI:** 10.3389/fpsyg.2023.1320703

**Published:** 2023-11-27

**Authors:** Samuel Ken-En Gan, Yusong Zeng, Zihan Wang

**Affiliations:** ^1^Applied Psychology and Discovery (APD), Joint Lab of Wenzhou-Kean University, Wenzhou, Zhejiang Province, China; ^2^James Cook University, Singapore, Singapore; ^3^College of Science and Technology, Wenzhou-Kean University, Wenzhou, Zhejiang Province, China; ^4^Wenzhou Municipal Key Lab for Applied Biomedical and Biopharmaceutical Informatics, Wenzhou, Zhejiang Province, China; ^5^Zhejiang Bioinformatics International Science and Technology Cooperation Center, Wenzhou- Kean University, Wenzhou, Zhejiang Province, China; ^6^Dorothy and George Hennings College of Science, Mathematics and Technology, Kean University, Union, NJ, United States

**Keywords:** workplace incivility, social anxiety, work engagement, well-being, mediation analysis, age, employee experience

## Abstract

The average working person spends between 35 and 60 h a week in the workplace, making it an influential place for mental well-being and a place for socioeconomic contribution. Workplace incivility can diminish positive mental health outcomes and negatively impact work engagement through increased social anxiety. To investigate this, 118 working adults in Singapore aged between 19 to 67 years old were recruited for a survey consisting of demographic questions, the Workplace Incivility Scale, the Brief DSM-5 Social Anxiety Disorder Severity Scale, and the Utrecht Work Engagement Scale-9 between November 2022 to April 2023. Correlational, regression, and mediation analysis showed workplace incivility scale scores to significantly predict social anxiety after controlling for covariates. This supports our hypothesis that employees exposed to workplace incivility would have higher social anxiety levels mediating work engagement after controlling for age and gender. The findings here show workplace incivility as a possible intervention target for social anxiety, in order to reduce negative impact on work engagement to improve employee experience and retention for organizations.

## Introduction

Workplace incivility (WPI) describes low-intensity deviant acts such as rudeness, condescending attitudes, and ignoring colleagues (Cortina et al., 2022). WPI can be ambiguous and are generally not malicious onslaughts like sexual harassment (Yao et al., 2022). WPI can be expressed as high-intensity workplace transgressions (e.g., physical intimidation) that aggravate debilitating mental health outcomes such as depression, anxiety, stress, emotional exhaustion, and lowered well-being (Schilpzand et al., 2016) and lead to negative consequences such as decreased job performance, lower productivity, work withdrawal behaviors, and turnover intentions (Vasconcelos, 2020) for organizations (Agarwal et al., 2023). In a 5-week study, significant stress was experienced by employees (*n* = 130) of a security firm in New South Wales (Australia) during the days in which they reported greater levels of WPI (Beattie and Griffin, 2014). Depression, higher levels of anger, and lowered self-esteem were associated with daily WPI in another 10-day longitudinal study of Swiss workers (*n* = 164) from various professional backgrounds (Adiyaman and Meier, 2022). WPI was also associated with lowered subjective well-being, increased headaches, sleep disturbances, and digestive problems in a cross-sectional study of nurses (*n* = 290) in a south-eastern US state (Sherrod and Lewallen, 2021) and for teachers (*n* = 341) in both government and private colleges in Jammu (India; Sood and Kour, 2022).

On the association between WPI and anxiety, a cross-sectional study involving postal workers (*n* = 950) in Canada, Geldart et al. (2018) reported that WPI was positively associated with anxiety, depression, hostility, and burnout after controlling for demographic and work factors. Similarly, a six-month longitudinal study on Romanian workers found that employees with trait anxiety reported higher bullying (Reknes et al., 2021). In China, junior nurses (*n* = 903) across 29 provinces showed that anxiety partially mediated WPI and job burnout (Shi et al., 2018). In contrast, telecommunications employees (n = 507) from six small- to medium-sized enterprises and companies in Pakistan (De Clercq et al., 2020) showed that anxiety mediated WPI and depersonalized behavior.

Cortina et al. (2017) found that women and younger workers were more prone to experiencing WPI compared to men and older workers. A significant negative medium-sized effect was found between WPI and age, but not gender (Han et al., 2022). In Singapore, men and younger workers experienced more WPI compared to women and older workers (Lim and Lee, 2011). Considering the significant negative Spearman correlation between social anxiety and civility (Cheok et al., 2020), there is an important role for civility in the workplace and society at large. The discrepancies in gender effect studies were likely due to local differences in perspectives on ethnic groups and biological sexes (McCord et al., 2018), with a notable decrease in biological effects over the years.

Collectively, the association between workplace social stressors and both employee health and workplace attitude/behavior were previously reported to be of medium effect size (r = −0.30, *p* < 0.001) from a meta-analysis (Gerhardt et al., 2021) of 555 studies. Thus, anxiety may play an indirect or mediating role between WPI and mental well-being.

For the organization, WPI negatively affected job performance and innovation (Jiang et al., 2019), conversely increasing turnover intentions (Namin et al., 2022). More subtly, knowledge-hiding can occur (Arshad and Ismail, 2018). Work withdrawal in the form of cyberloafing or spending time on the internet for non-work purposes notably increased with increased WPI among civil servants (*n* = 327) in Nigeria (Chine et al., 2019), with some employees spending precious time crafting retaliatory responses to rude emails (McCarthy, 2016).

Work engagement, which is defined as being immersed in work with vigor, dedication, and absorption (Schaufeli et al., 2006), is also negatively impacted. Examinations of decreased job satisfaction in Malaysian civil servants (Alias et al., 2020) and Taiwanese hospitality staff (Wang and Chen, 2020) found both co-worker and customer incivility to lead to reduced work engagement and job performance. Among frontline hotel employees in the Midwest USA (Im and Cho, 2021), supervisor WPI negatively correlated with employee engagement and self-efficacy, resulting in reduced service delivery.

Despite the vast literature on WPI, there are few mediation analyses investigating WPI and work engagement. One study on working adults in the United States with depression and/or bipolar disorder (*n* = 272) showed WPI and work engagement to be mediated through suicidal ideation among employees (Follmer and Follmer, 2021). Interestingly, in China, job insecurity mediated WPI and work engagement (Guo et al., 2022) without significant direct effects from WPI on work engagement.

To further investigate the effect of WPI on social anxiety and work engagement, this study investigates the hypotheses that: (1) WPI will positively and significantly predict social anxiety after controlling for the covariates of age and gender; (2) WPI will have an indirect negative impact on work engagement through social anxiety.

## Materials and methods

### Design

**Figure 1:**
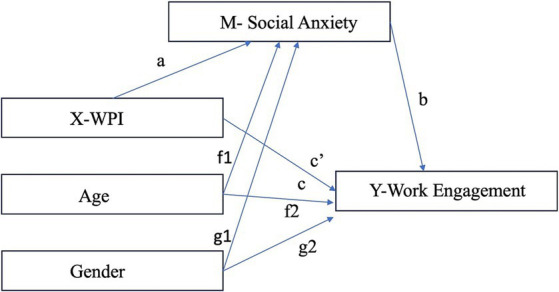
Conceptual diagram for the mediation process.

The study utilized a cross-sectional correlational study with the dependent variable as work engagement and the independent variable as WPI. The expected mediator was social anxiety, with age and biological sex as covariates. The proposed mediation process is illustrated in Figure 1.

### Participants

G*Power computation suggested a minimum of 85 participants for linear multiple regression (R2 deviation from zero) models: f2 = 0.095 small effect size, 0.05 two-tailed alpha, 0.80 power and 4 predictors (WPI, social anxiety, work, and covariates). The convenient recruitment strategy attracted 152 participants, but 34 were removed as they did not provide informed consent (*n* = 11) or did not answer all the questions in the scales (*n* = 23), resulting in a final number of 118 participants. The mean age (there were 5 missing data) was 33.7 ± 11.8 years (range between 19 to 67), consisting of 57% women, 42% men, and 1% non-binary. Ethnically, 79% were ethnic Chinese, 12% ethnic Indians, and 8% Malays/Others. Regarding education, 68% reported at least a bachelor’s degree or higher, 31% responded with College/Diploma, and other qualifications made up 1%. By industry, 89% of participants worked in the services industry, and 11% were based on goods. 54% of the participants were office-based, 38% in hybrid work arrangements, and 8% working from home.

The Workplace Incivility Scale (WIS; Cortina et al., 2001) for WPI measurement was modified to reflect “general employment” instead of the “Eight Circuit Court” and a “6-month” instead of a “5-year” retrospective recall period. The latter was modified for congruency with the period for the social anxiety scale. The WIS comprised seven questions: “Have you been in a situation where any of your superiors or co-workers have…” (e.g., “put you down or was condescending towards you?”). All item responses were on a 5-point Likert Scale (1 = Never to 5 = Most of the time). The final score was computed as a mean ranging from 1 to 5, with higher scores reflecting a higher WPI. The scale was previously used on the Singapore population by Lim and Lee (2011), which reported a Cronbach’s Alpha (α) of 0.91 (co-workers) to 0.92 (superiors). In the present study, α was 0.93, indicating excellent reliability.

The Brief DSM-5 Social Anxiety Disorder Severity Scale (SAD-6; Rice et al., 2021) had a time frame of the “past week” in the SAD-6 that was amended to the “past 6 months” in the present study. The amendment was unlikely to affect the measurement of social anxiety given that the SAD-6 is aligned with the DSM-5, which had a recommended span of 6 months to observe symptoms for social anxiety (Rice et al., 2021). The SAD-6 consists of six questions where participants rated the frequency of their feelings in social situations over the past 6 months (e.g., “Felt anxious, worried or nervous about social situations”). All items were on a 5-point Likert Scale (0 = Never to 4 = All the time). Higher final mean scores reflected higher levels of social anxiety. The SAD-6 previously had a Cronbach’s (α) of 0.95, which was identical to the present study, indicating excellent reliability.

The Utrecht Work Engagement Scale-9 (UWES-9; Schaufeli et al., 2006**)** consisted of nine questions where participants rated the degree of engagement they felt at work (e.g., “My job inspires me”). All items were on a seven-point Likert scale (0 = Never to 6 = Every Day), and the final mean score ranged from 0 to 6, with higher scores reflecting higher levels of work engagement. The scale was validated in a study involving Singapore working parents of children with disabilities (Stefanidis and Strogilos, 2020). The present study’s Cronbach’s (α) of 0.95 was the same as the original study’s 0.95.

### Procedure

Ethics approval was obtained from the James Cook University Ethics Committee (H8926). A Research Data Management Plan was registered.

Participants were recruited through convenience sampling via digital advertisements on the university bulletin board; the social media of the investigators, APD PsychVey (Liew et al., 2020), and Facebook Survey Exchange for thesis projects.^1^ Working adults aged 18 years and above were invited via a QR code/URL link, which brought them to the 10-min Qualtrics page online survey. The informed consent form had the information and clear instructions that the participants were free to end the survey at any time without prejudice but that once the anonymous responses were submitted, they could not be identified for deletion. Participant data without complete informed consent were removed. Participants were asked to respond to the scales mentioned above (WIS, SAD-6 and UWES-9) and some demographic questions modified from previous surveys (Gan et al., 2003; Chew et al., 2016; Wan et al., 2021; Gan et al., 2023) about age, biological sex, race, education, industry, occupation-type (e.g., services), and work location (e.g., home). The survey ended with a debriefing about the study’s aims and sources for psychological services if necessary. There were no benefits provided for the participation.

## Results

### Assumption testing

Major regression assumptions (mentioned in Tabachnick and Fidel, 2019; Pallant, 2020) were met in the present study. Firstly, the residuals were deemed to be independent as the data points were not correlated with each other (Durbin-Watson statistic = 2.01, falling within the acceptable ranges of 1 and 3). Secondly, there was homoscedasticity in residuals as indicated by the elliptical scatter plot of the regression standardized predicted values against the regression standardized residuals. Thirdly, the errors were normally distributed: (1) The histogram of the errors appeared somewhat bimodal though approximately normal, i.e., not skewed; (2) The normal P–P plot of the residuals showed some deviation from the plot’s predicted straight line but was generally normal; and (3) Both the Kolmogorov–Smirnov and Shapiro–Wilk tests of the unstandardized residuals were not statistically significant (*p* = 0.200 and *p* = 0.235, respectively). Fourthly, there were linear relationships between the dependent variable (work engagement) and each of the continuous variables (WPI, social anxiety, and age) from the scatter plots. Next, multicollinearity was not evident as zero-order correlation coefficients among the independent variables were below 0.70, VIF was under 5, and Tolerance was above 0.20. Finally, there was no undue influence as Cook’s Distance for all data points was below 1.

### Descriptive statistics

The descriptive statistics are shown in Table 1. The mean WPI score of 1.94 ± 0.88 was close to the WIS Likert score of 2 (i.e., seldom experience incivility at the workplace). Comparatively, 89% (all the mean scores of WPI are above 1) showed that most participants experienced some form of WPI (score above 1). This is akin to the 91% prevalence reported by Lim and Lee (2011). Thus, while there is a high prevalence of workplace incivility, the intensity is relatively low. The mean social anxiety score of 1.14 ± 0.98 was close to the SAD-6 Likert score of 1 in the SAD-6 (i.e., occasionally experienced social anxiety). When the SAD-6 was computed as a sum of 6.82 ± 5.9 out of 40, it was found to be similar to the 10.8 ± 8.89 score reported for a community sample by Rice et al. (2021). The mean work engagement score of 3.14 ± 1.35 was close to the UEWS-9 score of 3 (i.e., “sometimes or a few times a month” felt total engagement with their work). Comparatively, this is one Likert scale point lower than that reported by Stefanidis and Strogilos (2020) finding that parents of children with special needs often felt engaged with their work, possibly given their supportive environment.

**Table 1:** Descriptive statistics and correlation coefficients.

	Measure	M	SD	1	2	3	4
1	WPI	1.94	0.88	-	-	-	
2	Social Anxiety	1.14	0.98	0.55^***^	-	-	
3	Work Engagement	3.14	1.35	−0.414^***^	−0.55^***^	-	
4	Age	33.73	11.78	−0.24^*^	−0.38^***^	0.43^***^	
5	Gender	-	-	−0.11	−0.04	0.07	0.03

M = mean, SD = Standard Deviation, Gender (−1 = male, 0 = non-binary, 1 = female). *p < 0.05. **p < 0.01. ***p < 0.001.

### Correlational analysis

A significant, positive relationship was found between WPI and social anxiety (r = 0.55, *p* < 0.001) and between age and work engagement (r = 0.43, *p* < 0.001), whereas there was a significant, negative association between age and WPI (r = −0.24, *p* = 0.01); WPI and work engagement (r = −0.414, *p* < 0.001); social anxiety and work engagement (r = −0.55, *p* < 0.001); and age and social anxiety (r = −0.38, *p* < 0.001). Biological sex did not significantly correlate with WPI, social anxiety, work engagement, or age.

Generally, higher levels of WPI were associated with higher levels of social anxiety but lower work engagement.

### Hierarchical regression

To measure the impact of WPI on social anxiety (after controlling for age and biological sex), hierarchical regression analysis showed that WPI accounted for 20.3% of the variation in social anxiety [ΔR2 = 0.203, ΔF(*F* (1,108) =) = 33.67, *p* < 0.001] with a medium effect size (f2 = 0.25).

Table 2 shows the hierarchical regression of WPI affecting social anxiety and covariates (age and biological sex) on work engagement, with Model 1 consisting of the covariates age and gender that accounted for 18.90% of the variation in work engagement (ΔR2 = 0.19, ΔF (2,110) = 12.92, p < 0.001) with a medium effect size (f2 = 0.23). In Model 2, WPI was introduced after controlling for age and gender, contributing to a further 12.6% of the variation in work engagement [ΔR2 = 0.13, ΔF (1,109) = 20.94, *p* < 0.001] with a medium effect size (f2 = 0.15). In Model 3, social anxiety was introduced. After controlling for age, gender and WPI contributed a further 6% of the variation [ΔR2 = 0.06, ΔF (4,108) = 10.63, *p* < 0.002]. This final model accounted for about 38.2% of the variation in work engagement [R2 = 0.38, ΔF (4,108) = 16.66, *p* < 0.001] with a large effect size (f2 = 0.60). Social anxiety had the most significant impact (β = −0.31, *p* = 0.002), followed by age (β = 0.25, *p* = 0.003) and then WPI (β = −0.23, *p* = 0.01) with biological sex showing *p* > 0.05. Social anxiety weakened the contribution of WPI (β of WPI decreased from −0.37 to −0.23), suggesting a significant partial mediating effect.

**Table 2:** Hierarchical Regression Model.

Variable	Model 1			Model 2			Model 3		
	B	SE	β	B	SE	β	B	SE	β
Age	0.05	0.01	0.43***	0.04	0.00	0.34***	0.03	0.01	0.25**
Gender	0.24	0.24	0.08	0.1	0.21	0.03	0.12	0.20	0.04
WPI				−0.60	0.13	−0.37***	−0.37	0.15	−0.23*
Social Anxiety							−0.43	0.13	−0.31**
R2			0.19			0.32			0.38
ΔR2			0.19***			0.13***			0.06**

B: unstandardized regression coefficient; β: standardized regression coefficient, SE: Standard Error.

### Mediation analysis

**Figure 2:**
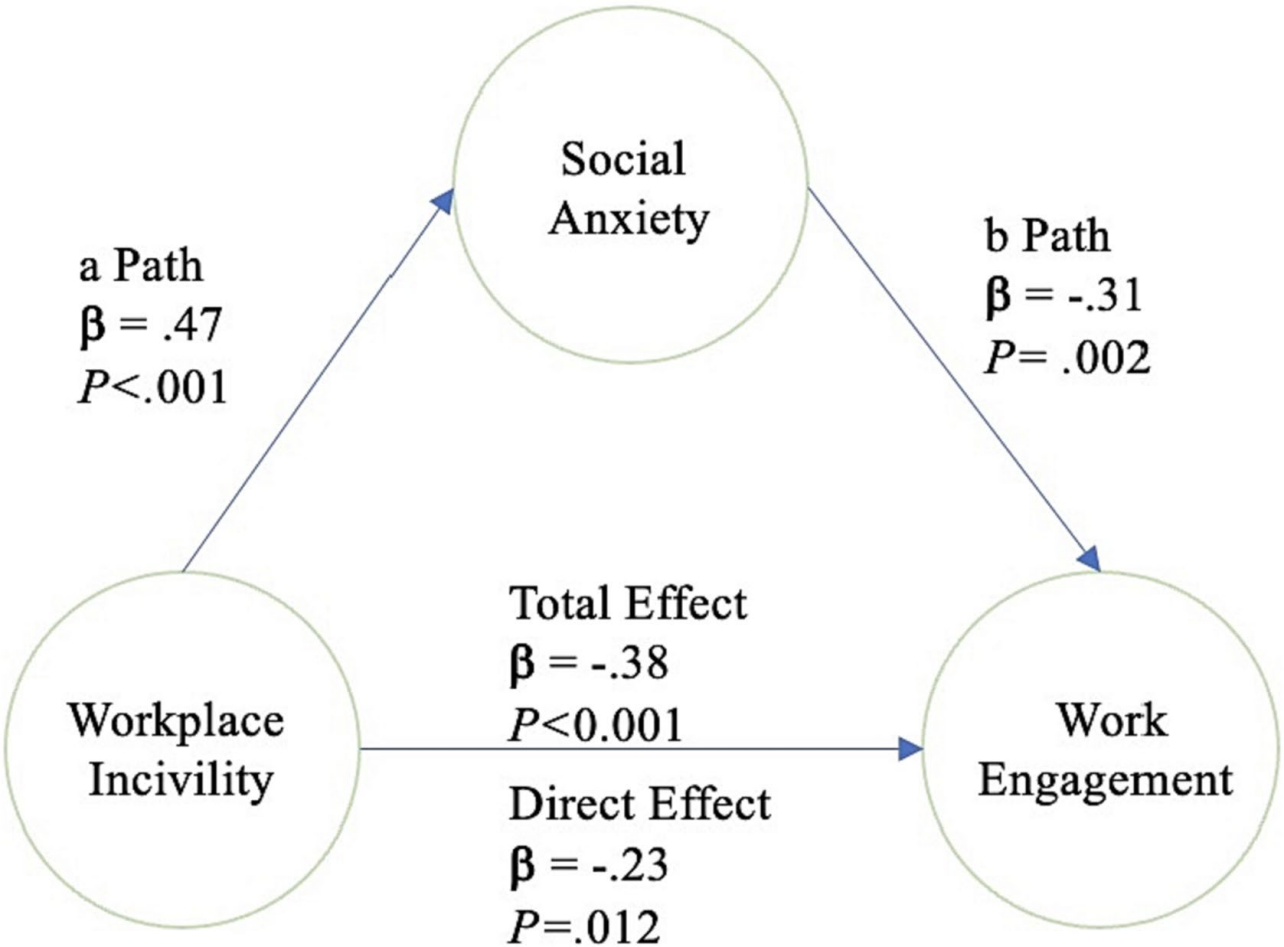
Simple mediation model.

Mediation analysis was performed using Model 4 of Hayes (2018) PROCESS macro (v.4.2) for SPSS, reporting 95% confidence intervals (CI) for the indirect effect based on percentile bootstrapping with 5,000 samples. The results are presented in Table 3 and Figure 2.

**Table 3:** Path coefficient for simple mediation model.

Effect	Path	B	LLCI	ULCI	SE	β	*p*
a	WPI → Social anxiety	0.55	0.36	0.73	0.09	0.47	0.000
b	Social anxiety → Work engagement	−0.42	−0.68	−0.17	0.13	−0.31	0.002
Total effect: c	WPI → WE	−0.60	−0.86	−0.34	0.13	−0.38	0.000
Direct effect: c’	WPI → WE	−0.36	−0.66	−0.08	0.15	−0.23	0.012
Indirect effect: ab	WPI → SA → WE	−0.23	−0.41	−0.08	0.08	−0.15	-
f1	Age → SA	−0.02	−0.04	−0.01	0.01	−0.27	0.001
f2	Age → WE	0.03	0.01	0.05	0.01	0.25	0.003
g1	Gender → SA	0.03	−0.26	0.33	0.15	0.02	0.83
g2	Gender → WE	0.12	−0.29	0.52	0.20	0.04	0.57

WPI, workplace incivility; SA, social anxiety; WE, work engagement; B, unstandardized coefficient; LLCI, lower-level CI; ULCI, Upper-Level CI; SE, standard errors; β, standardized coefficient; p, probability.

Hypothesis 1: WPI would positively and significantly predict social anxiety after controlling for covariates age and biological sex.

After controlling for covariates, age was significantly negatively associated with social anxiety. Biological sex did not show significant associations. Controlling for both age and gender, WPI was significantly and positively associated with social anxiety (a path) with a medium effect size [Δ(ΔR2 = 0.20)]. Thus, the first hypothesis of this study was accepted.

Hypothesis Testing 2: WPI will indirectly negatively impact work engagement through social anxiety.

WPI significantly negatively affected work engagement (c path), after controlling for age and biological sex. Social anxiety was significantly negatively associated with work engagement (b path) after controlling for WPI, age, and biological sex. Thus, the indirect effect of WPI on work engagement through social anxiety (ab path) was shown to be significantly negative. The direct effect of WPI on work engagement, controlling for social anxiety and covariates, was also significantly negative (c’ path). Therefore, WPI had a negative effect on work engagement, and this relationship could be explained through the mediating effect of social anxiety. Specifically, social anxiety partially mediated between WPI and work engagement because the c’ path remained statistically significant. Thus, the second hypothesis of the study was also accepted.

### Simple mediation model

Small, medium, and large effect sizes (f2) were based on values of 0.02, 0.15, and 0.35, respectively (Cohen, 1988). Hayes PROCESS macro was used to estimate the mediation effect of social anxiety (Hayes, 2018).

## Discussion

This study aimed to investigate if WPI could positively predict social anxiety and if it indirectly negatively impacted work engagement through social anxiety (partial or complete mediation). The analysis supported accepting both hypotheses, given that WPI was positively associated with social anxiety after controlling for covariates and its negative impact on work engagement, partially mediated by social anxiety.

Upon controlling for covariates, WPI showed a medium effect in predicting social anxiety, akin to the medium effect reported between workplace social stressors and employee health in a meta-analysis (Gerhardt et al., 2021). Employees who experienced higher levels of WPI also had higher levels of social anxiety. The findings here were also congruent with other studies finding the negative impact of WPI on stress (Beattie and Griffin, 2014), depression (Adiyaman and Meier, 2022), lower subjective well-being (Sherrod and Lewallen, 2021), lower psychological well-being (Sood and Kour, 2022), rumination after work (Vahle-Hinz, 2019), and anxiety (Geldart et al., 2018).

Our findings here updated the literature on WPI and social anxiety, where most studies to date utilized scales developed before 1985, and we further found social anxiety to be a mediating factor. While Lim and Lee (2011) did not find a significant association between anxiety with either co-worker or supervisor-initiated WPI in their Singapore employees, the study utilized a different scale to capture a different form of anxiety other than social anxiety. Nonetheless, the related congruency with another finding by Cheok et al. (2020) of an association between social anxiety and civility may reflect changing perspectives or work environments in Singapore workplaces in the early 2010s and the 2020s.

The accepted second hypothesis that the negative impact of WPI on work engagement was partially mediated through social anxiety (partial mediation) after controlling for age and gender, agreed with the meta-analysis study reporting a medium effect between social stressors and workplace attitudes/behavior (Gerhardt et al., 2021). Employees who experienced more WPI were more likely to have aggravated social anxiety that would erode their work engagement in the organization. Given that the mediation effect was only partial, it is likely that there were other factors that contributed to the work engagement that could be investigated in future studies.

The negative impact of WPI in the workplace, such as on turnover intentions (Namin et al., 2022), job performance (Jiang et al., 2019), and work withdrawal (Bernard and Joe-Akunne, 2019) in many employee types such as civil servants in Malaysia (e.g., Alias et al., 2020), hospitality industry employees in Taiwan (Wang and Chen, 2020), and the USA (e.g., Im and Cho, 2021), showed the general far-reaching effects of WPI. The specific findings in this study on WPI and work engagement could further incorporate the findings that job security mediated WPI and work engagement (Guo et al., 2022), with the explanation that WPI, especially by superiors, can negatively impact job security. Given that social anxiety was a partial factor, job security could also be studied together alongside suicidal ideation (Follmer and Follmer, 2021) to further make sense of other earlier studies on the role of anxiety or its various types (Shi et al., 2018; De Clercq et al., 2020).

Our analysis showed that age was negatively correlated with WPI in support of literature (Cortina et al., 2017; Han et al., 2022) and that employees generally experienced less WPI as they grew older. Younger and less experienced workers may face more WPI, or some form of work hazing may be present in many workplaces. Notably, we did not find any biological sex effects with WPI, despite contradicting older literature finding that women (Cortina et al., 2017) or men (Lim and Lee, 2011) experienced more WPI, which lends support to more recent findings (McCord et al., 2018) that biological sex and ethnic biases have diminished over the years, at least as found in our sample and in some of our recent studies (Ng et al., 2018; Gan et al., 2023).

This study is limited in that it did not take into account the nature of the work of the participants, which would naturally affect the amount of human interaction that may correlate to workplace incivility exposure. In addition, the study was carried out during the end of the COVID-19 pandemic, which would have an impact on social anxiety and even communication in the workplace. It would also be interesting to see if background music, which previously could reduce mathematical anxiety (Gan et al., 2016), and affect learning (Chew et al., 2016), could be used to alleviate social anxiety, especially when there had been attempts to manipulate emotions through music in retail (Choo et al., 2021). Such gaps may be useful in exploring workplace environments as a form of intervention for the ill-effects of workplace civility and social anxiety.

## Conclusion

Our mediation study showed that social anxiety is a new mediator of working engagement and work place incivility, renewing the findings of Lim and Lee’s (2011) work. Furthermore, our study also demonstrated that organizations need to be mindful of WPI, which can erode work engagement. Displayed social anxiety may be a useful early symptom of the general mental well-being of the employee in the workplace, and lower levels of work engagement may be a manifestation of underlying issues relating to psychological well-being and mistreatment at work. It would be important for these to be addressed early for improved general employee experience in which managers may choose to incorporate more open communication channels and penalize overt displays of incivility among colleagues. More support may be necessary for the younger employees that experienced greater WIP, although all these measures would need to take into consideration the organizational culture and nature of the work, to which further investigations would help provide a more tailored approach.

## Data availability statement

The raw data is available from the corresponding author upon reasonable request.

## Ethics statement

The studies involving humans were approved by James Cook University Ethics Committee (H8926). The studies were conducted in accordance with the local legislation and institutional requirements. The participants provided their written informed consent to participate in this study.

## Author contributions

SG: Conceptualization, Data curation, Project administration, Supervision, Writing – original draft, Writing – review & editing. YZ: Data curation, Formal Analysis, Validation, Writing – review & editing. ZW: Writing – review & editing.
